# A Detailed Data-Driven Network Model of Prefrontal Cortex Reproduces Key Features of *In Vivo* Activity

**DOI:** 10.1371/journal.pcbi.1004930

**Published:** 2016-05-20

**Authors:** Joachim Hass, Loreen Hertäg, Daniel Durstewitz

**Affiliations:** 1 Department of Theoretical Neuroscience, Bernstein-Center for Computational Neuroscience, Central Institute of Mental Health, Medical Faculty Mannheim of Heidelberg University, Mannheim, Germany; 2 Modelling of Cognitive Processes, Berlin Institute of Technology and Bernstein Center for Computational Neuroscience Berlin, Germany; Indiana University, UNITED STATES

## Abstract

The prefrontal cortex is centrally involved in a wide range of cognitive functions and their impairment in psychiatric disorders. Yet, the computational principles that govern the dynamics of prefrontal neural networks, and link their physiological, biochemical and anatomical properties to cognitive functions, are not well understood. Computational models can help to bridge the gap between these different levels of description, provided they are sufficiently constrained by experimental data and capable of predicting key properties of the intact cortex. Here, we present a detailed network model of the prefrontal cortex, based on a simple computationally efficient single neuron model (simpAdEx), with all parameters derived from *in vitro* electrophysiological and anatomical data. Without additional tuning, this model could be shown to *quantitatively* reproduce a wide range of measures from *in vivo* electrophysiological recordings, to a degree where simulated and experimentally observed activities were statistically indistinguishable. These measures include spike train statistics, membrane potential fluctuations, local field potentials, and the transmission of transient stimulus information across layers. We further demonstrate that model predictions are robust against moderate changes in key parameters, and that synaptic heterogeneity is a crucial ingredient to the quantitative reproduction of *in vivo*-like electrophysiological behavior. Thus, we have produced a physiologically highly valid, in a quantitative sense, yet computationally efficient PFC network model, which helped to identify key properties underlying spike time dynamics as observed *in vivo*, and can be harvested for in-depth investigation of the links between physiology and cognition.

## Author Summary

Computational network models are an important tool for linking physiological and neuro-dynamical processes to cognition. However, harvesting network models for this purpose may less depend on how much biophysical detail is included, but more on how well the model can capture the *functional* network physiology. Here, we present the first network model of the prefrontal cortex which has not only its single neuron properties and anatomical layout tightly constrained by experimental data, but is also able to *quantitatively* reproduce a large range of spiking, field potential, and membrane voltage statistics obtained from *in vivo* data, without need of specific parameter tuning. It thus represents a novel computational tool for addressing questions about the neuro-dynamics of cognition in health and disease.

## Introduction

The prefrontal cortex (PFC) is a key structure in higher-level cognitive functions, including working memory, rule and concept representation and behavioral flexibility [1–6], and has been linked to impairments of these functions in psychiatric disorders like schizophrenia [7–10] or attention-deficit/hyperactivity disorder [11]. Our understanding of the computational and dynamic mechanisms underlying these cognitive functions, their neuromodulation, and their aberrations in psychiatric disorders, is still very limited, however.

Computational network models are a highly valuable tool for driving forward such an understanding, as data from many different levels of experimental analysis can be integrated into a coherent picture. With respect to psychiatric conditions, it is of particular importance that models incorporate sufficient biological detail and exhibit physiological validity in order to serve as explanatory tools. Psychiatric conditions like schizophrenia are characterized by a multitude of abnormalities in diverse cellular and synaptic properties, transmitter systems, and neuromodulatory input [7–10]. Moreover, pharmacological treatment options target the neurochemical and physiological level, yet they are supposed to change functionality at the behavioral and cognitive level. It is thus crucial to gain insight into the explanatory links between behavioral functions and the underlying neurobiological “hardware”, a task that requires sufficient physiological detail in the model specification, in particular realistic assumptions about anatomical structure and cell type diversity.

Ultimately, the physiological validity of a computational model ought to be reflected in the degree to which it can reproduce and predict detailed aspects of the neural activity observed *in vivo*. That is, from a statistical perspective, one may define a good, physiologically valid model as one that accurately (i.e., quantitatively) captures distributions compiled from the electrophysiological activity (spiking, field potentials, membrane voltages) produced by networks *in vivo*, but not necessarily as one that captures every detail of membrane biophysics or receptor kinetics. In our perception, such requirements are currently not met even by sophisticated cortical network models which do include a lot of biophysical detail [12–14], as these are often only loosely compared to *in vivo* data or test only specific aspects of those.

In this work, we present a computational network model of the PFC which has high physiological validity and predictivity both at the single-neuron- (*in vitro*) and at the network- (*in vivo*) level, yet is still simple enough to be computationally tractable. Its anatomical structure, neural, and synaptic properties are completely derived from the experimental literature and our own experimental data. The activity of the network is compared with a range of statistics derived from *in vivo* data, including spike trains, local field potentials, and membrane potential fluctuations. The model turns out to reproduce these data *quantitatively*, and also exhibits robustness with respect to moderate changes in parameters.

## Results

### Key features of the prefrontal cortex model

The network model introduced in Materials and Methods aims to combine computational tractability with physiological validity. This balance is achieved by embedding a simple, reduced two-dimensional single neuron model into a realistic network architecture that is derived from the experimental literature. All model parameters were directly estimated from our own *in vitro* data and the experimental literature (see Materials and Methods for details), and no specific parameter tuning was necessary to bring the network model closer to *in vivo*-like behavior.

**Fig 1:**
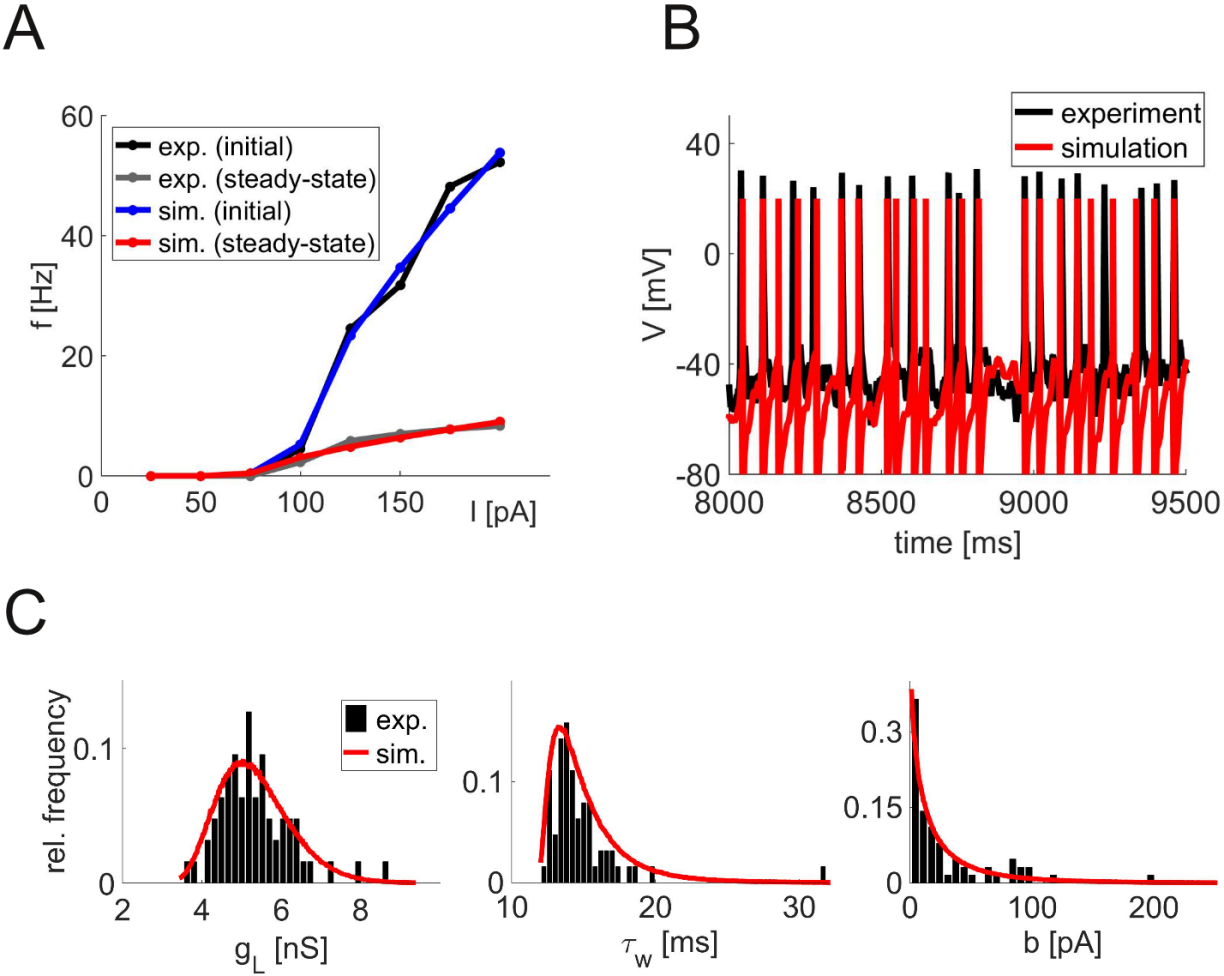
Single neuron recordings and model fitting. (A) Example of the initial (upper curve) and steady-state (lower curve) input-output relation (f-I curve) of a single neuron. Black and gray curves show experimental data, red and blue curves indicate the simpAdEx model fits. (B) Voltage trace from a slice recording of a prefrontal cortical layer 5 pyramidal cell (black) and from the corresponding model cell (red) in response to the same fluctuating input current. The same neuron model and parameters as in Panel A were used [15]. (C) Examples of parameter distributions obtained from fitting model neurons to electrophysiologically recorded cells. Histograms (black) and derived parameter distributions used for network specification (red) illustrating parameters with an approximately Gaussian (*g*_*L*_, left), Gamma (*τ*_*w*_, middle), and exponential distributional form (*b*, right).

At the single-cell level, the network is based on an approximation (simpAdEx [15]) to the adaptive exponential integrate-and-fire model (AdEx [16]) which yields closed-form expressions for instantaneous and steady-state firing rates, thus allowing for fast and fully automatized fitting to f-I and V-I curves from physiologically recorded cells (Fig 1A). We had shown previously that this cell model is able to accurately predict spike times of recorded neurons driven by *in vivo*-like fluctuating currents not used for model fitting [15] (Fig 1B) and, like the full AdEx [17], can generate a wide range of spike patterns. *In vitro* recordings from ∼200 L2/3 and L5 pyramidal cells, fast-spiking and bitufted interneurons from the medial PFC of adult rodents were used to generate a distribution of model cells that reflects the diversity of neurons in the real PFC (see Materials and Methods for details). The resulting model parameters (Table 1) follow broad distributions (Fig 1C), mostly of Gaussian shape, with the exception of Δ_*T*_ and *τ*_*w*_ which are best described by a Gamma distribution, and *b* which follows an exponential distribution (red curves in Fig 1C indicate distributions from which model parameters were drawn).

**Table 1:** Neuron parameters.

parameter	PC L2/3	FS	BT	MC	PC L5
*C* (pF)	164.96 (59.11)	59.58 (10.59)	79.36 (14.83)	81.12 (28.96)	251.81 (82.61)
*g*_*L*_ (nS)	7.04 (1.72)	5.34 (0.91)	3.99 (0.51)	2.98 (0.55)	7.62 (2.09)
*E*_*L*_ (mV)	-85.00 (5.40)	-85.15 (5.81)	-84.63 (4.71)	-72.20 (7.64)	-80.57 (6.71)
Δ_*T*_ (*mV*)	21.44 (6.42)	19.58 (8.50)	19.02 (4.08)	22.30 (10.44)	24.47 (5.96)
*τ*_*w*_ (ms)	121.78 (41.19)	15.15 (2.71)	43.56 (21.89)	60.13 (15.05)	107.48 (64.08)
*b* (pA)	7.29 (6.80)	34.87 (37.88)	6.65 (7.19)	5.37 (5.78)	8.27 (12.66)
*V*_r_ (mV)	-118.20 (38.14)	-90.16 (15.16)	-152.67 (49.15)	-55.89 (9.65)	-69.98 (14.45)
*V*_th_ (mV)	-52.40 (5.43)	-58.79 (9.82)	-59.95 (4.67)	-38.01 (6.03)	-48.69 (7.18)
*V*_up_ (mV)	-45.91 (7.22)	-51.01 (5.59)	-55.46 (4.39)	-36.94 (2.55)	-44.12 (7.28)

Mean and standard deviation of the parameters of the simpAdEx model for the five different neuron types used in the network (PC: Pyramidal cell, FS: Fast-spiking interneuron, BT: Bitufted interneuron, MC: Martinotti cell)

**Fig 2:**
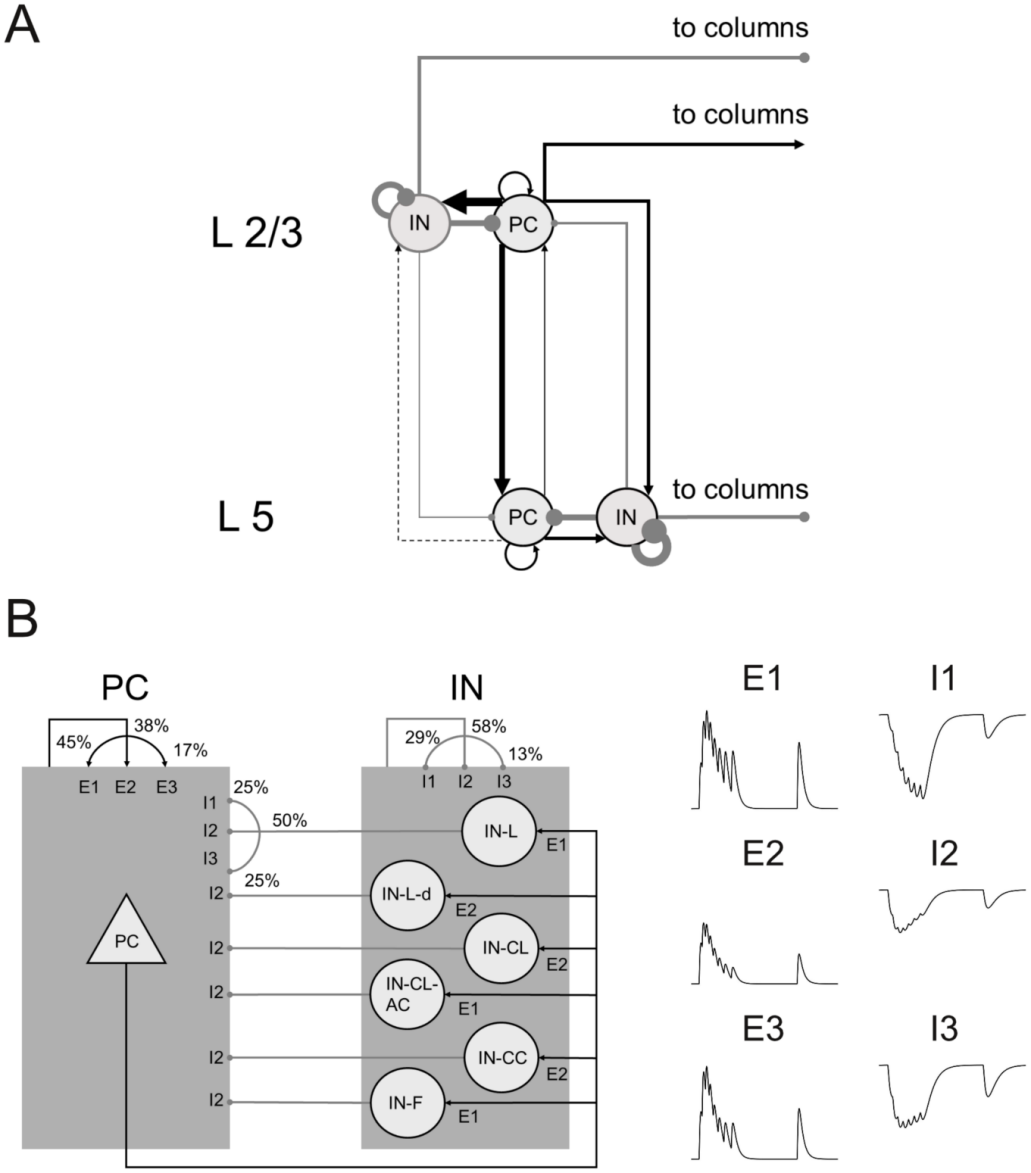
Anatomical and synaptic properties. (A) Laminar structure of a single network column. Arrow widths represent relative strength of connections (black: excitatory, gray: inhibitory), i.e. the product of connection probability and synaptic peak conductance. (B) Left panel: Distribution of three different short-term plasticity types over different combinations of pre- and postsynaptic neuron types. Arrows from or to one of the shaded blocks (rather than from or to a single neuron type) denote connection types that are identical for all excitatory (PC) or inhibitory (IN) neurons. Where all three types are drawn, they are randomly distributed over all synapses between these two neuron types according to the probabilities given in the figure. Right panel: Illustration of the postsynaptic potential in response to a series of presynaptic spikes for three types of short-term synaptic plasticity for excitatory (E1 to E3) and inhibitory synapses (I1 to I3).

Anatomically, the network is divided into two laminar components, representing the superficial layers L2/3 and deep layer L5 (Fig 2A). Neurons are distributed over the five cell types in each layer based on estimates from the literature (Table 2). The neurons are randomly connected with different connection probabilities *p*_con_ for each pair of cell types according to the literature [18–27], including local clusters of higher connectivity [28, 29]. The neurons are assumed to be organized in a single column and horizontal spatial distance is not taken into account. However, all neurons receive a *constant* background current (i.e., without fluctuations) that represents synaptic connections from outside the network, both within and outside the same column (see section “Admissable and realistic range of input currents” below). Since these currents were constant, all irregularity was produced intrinsically within the simulated network.

**Table 2:** Cell numbers.

Layer	PC	IN-L	IN-CL	IN-CC	IN-F
*L*2/3	47%	3.1%	2.6%	2.6%	2.1%
*L*5	38%	0.5%	0.5%	1.8%	1.8%

Relative numbers of cells for each type. PC: pyramidal cell, IN: interneuron, see Materials and Methods for interneuron subtypes.

Neurons are connected by conductance-based synapses (AMPA, GABA_A_ and NMDA) with kinetics estimated from electrophysiological data, short-term synaptic plasticity [30] that is matched to the types of the connected neurons [31, 32], synaptic delays and random failure of synaptic transmission [33–36]. Distributions of synaptic weights (log-normal [37]) and delays (Gaussian) were extracted from the literature (Table 3). The average connection strength (connectivity *p*_con_ times synaptic peak conductance *g*_max_) between pyramidal cells and interneurons in the different columns and layers is indicated by the width of the arrows in Fig 2A.

**Table 3:** Synaptic parameters.

pre	post	*p*_con_	*g*_max_ [mV]	*τ*_*D*_ [ms]
PC L2/3	PC L2/3	0.139	0.84 (0.49)	1.55 (0.31)
PC L2/3	PC L5	0.233	0.95 (0.39)	1.91 (0.17)
PC L5	PC L2/3	0.045	0.84 (0.28)	2.75 (0.18)
PC L5	PC L5	0.081	0.88 (0.67)	1.56 (0.44)
PC L2/3	IN-L L2/3	0.325	1.34 (1.09)	0.96 (0.25)
PC L2/3	IN-CL L2/3	0.159	0.47 (0.20)	0.96 (0.25)
PC L2/3	IN-F L2/3	0.290	0.25 (0.20)	0.96 (0.25)
PC L2/3	IN-L L5	0.087	0.77 (0.86)	1.18 (0.13)
PC L2/3	IN-CL L5	0.080	0.27 (0.16)	1.18 (0.13)
PC L2/3	IN-F L5	0.150	0.14 (0.16)	1.18 (0.13)
PC L5	IN-K L2/3	0.188	1.52 (0.63)	1.05 (0.08)
PC L5	IN-CL L2/3	0.092	0.53 (0.12)	1.05 (0.08)
PC L5	IN-F L2/3	0.168	0.28 (0.12)	1.05 (0.08)
PC L5	IN-L L5	0.333	1.74 (1.12)	0.60 (0.20)
PC L5	IN-CL L5	0.080	0.88 (0.70)	0.60 (0.20)
PC L5	IN-F L5	0.362	0.28 (0.30)	0.60 (0.20)
IN-L L2/3	PC L2/3	0.466	2.30 (1.98)	1.25 (0.18)
IN-CL L2/3	PC L2/3	0.301	0.13 (0.48)	1.25 (0.18)
IN-F L2/3	PC L2/3	0.710	1.91 (3.83)	1.25 (0.18)
IN-L L2/3	PC L5	0.217	1.07 (0.92)	1.54 (0.10)
IN-CL L2/3	PC L5	0.140	0.06 (0.22)	1.54 (0.10)
IN-F L2/3	PC L5	0.330	0.89 (1.78)	1.54 (0.10)
IN-L L5	PC L2/3	0.039	0.10 (0.01)	1.44 (0.04)
IN-CL L5	PC L2/3	0.027	0.04 (0.01)	1.44 (0.04)
IN-F L5	PC L2/3	0.040	0.07 (0.06)	1.44 (0.04)
IN-L L5	PC L5	0.274	0.69 (0.10)	0.82 (0.09)
IN-CL L5	PC L5	0.173	0.30 (0.05)	0.82 (0.09)
IN-F L5	PC L5	0.282	0.50 (0.40)	0.82 (0.09)
IN L2/3	IN L2/3	0.250	1.35 (0.35)	1.10 (0.40)
IN L5	IN L5	0.600	1.35 (0.35)	1.11 (0.40)

Mean and standard deviation of the parameters of the synapses connecting the different pre- and postsynaptic neuron types (*p*_con_: connection probability, *g*_max_: peak conductance, *τ*_*D*_: transmission delay. Cross-column interneurons (IN-CC) have the same parameters as local interneurons (IN-L), as they are both basket cells).

Wherever possible, we used data from the rodent prefrontal cortex, or at least agranular cortices such as the motor cortex, which in rodents shows a similar layered anatomy as the PFC. Apart from the missing granular layer 4, specific features of the rodent PFC that are modeled here include an increased fraction of reciprocal compared to unidirectional connections [32], longer NMDA time constants than in other areas [38, 39], and a uniquely prefrontal distribution of short-term synaptic plasticity properties for connections among pyramidal cells [32].

### Reproduction of *in vivo* activity

To assess whether the network model can reproduce the dynamics of real prefrontal neurons *in vivo*, we compared measures computed from the model with those from electrophysiological data, as well as with a number of findings from the literature. Unless otherwise stated, we simulate a single column with 1000 neurons and apply a constant DC current of 250 pA to all pyramidal cells and 200 pA to all interneurons. These currents are the only parameters that are not directly obtained from experimental data. As discussed below, appropriate values for these currents were derived by inferring from lumped-population input simulations the amount of current produced by a network of realistic size, set up with the very same structure as the explicitly modeled network.

**Fig 3:**
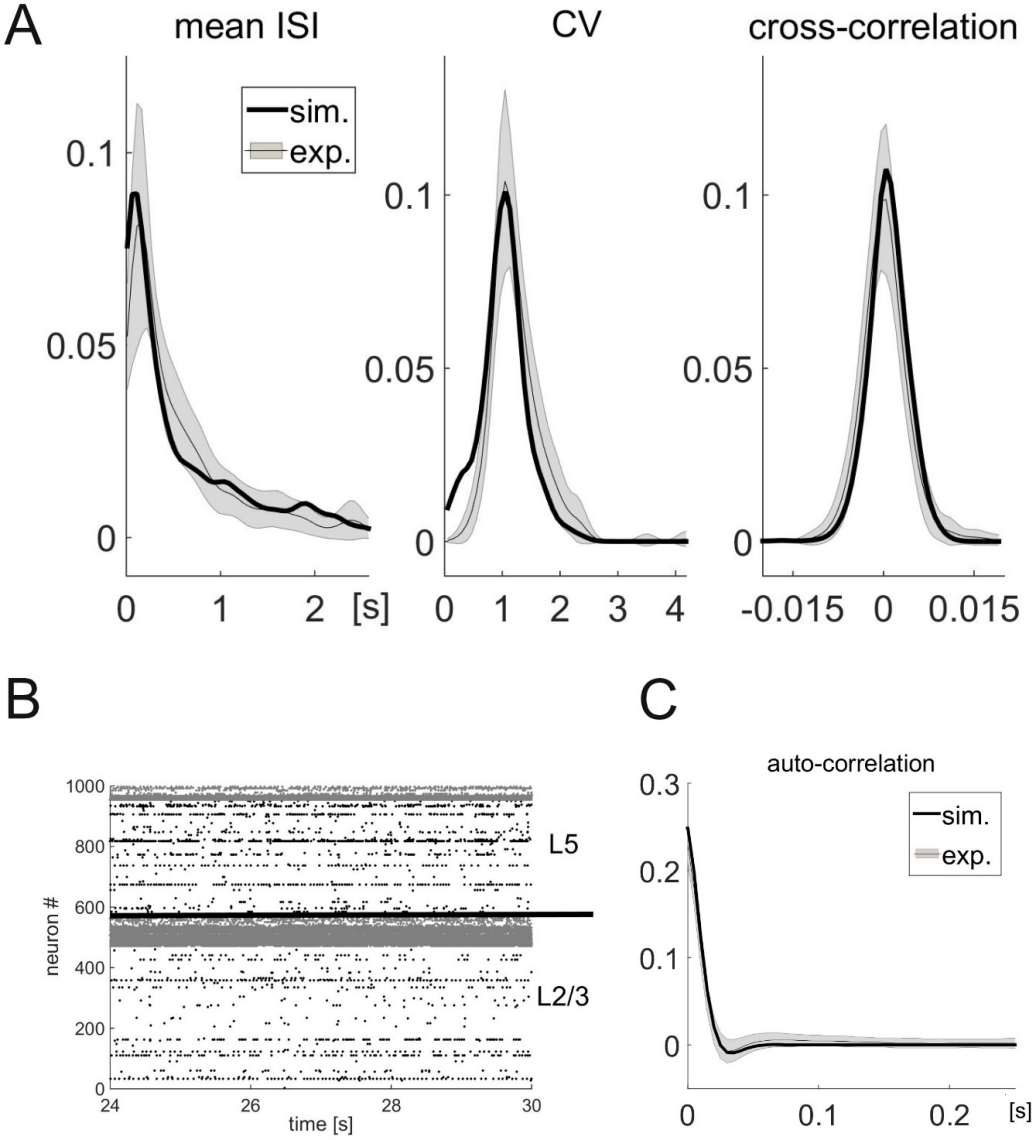
Spiking statistics of simulated PFC model networks. (A) Comparison of relative frequency histograms for three different spike time statistics between recordings from an *in vivo* experiment (gray, see text for details) and from the simulation with input currents *I*_ex_ = 250 pA, *I*_inh_ = 200 pA (black). The shaded region represents the mean ± the SEM at each point of the experimental distribution. (B) Raster plot of the spike times over the last six seconds of the simulation. The two layers (L2/3 and L5) are separated by a black line, pyramidal cells (PC) are in black, interneurons (IN) in gray. (C) Auto-correlation function of the inter-spike intervals of the experimental recordings (gray) and the network simulations (black).

**Spike-train statistics.** All experimentally recorded spike trains (kindly provided by Dr. Christopher Lapish, Indiana University Purdue University, Indianapolis, see [40] for details) were first segregated into statistically stationary segments to yield estimates of spike train statistics that reflect *in vivo* baseline activity, free from task-related responses (not modeled here) or other potential confounds [41]. For consistency, the same procedure was applied to the simulated spike trains, although, strictly, these were stationary by simulation setup. From all jointly stationary segments, the mean 〈ISI〉, coefficient of variation C_*V*_, and autocorrelation function of the inter-spike intervals (ISIs) were computed for each individual spike train, as well as the zero-lag cross-correlation CC(0) between pairs of neurons (Fig 3).

The *in vivo* data show very low zero-lag cross-correlations between neuron pairs (2.4 ⋅ 10^−4^ ± 2.5, mean ± SD) and C_*V*_ s near one (1.04 ± 0.33), consistent with the proposal of an “asynchronous-irregular” (AI) state of cortical dynamics (although the correlations theoretically proposed for the AI state are usually even at least one order of magnitude larger than obtained here [42]). The average single-cell ISIs follow a monotonically decreasing distribution with a mean comparable in size to the standard deviation (570 ± 610 ms), but with a heavy tail that is better described by a log-normal or beta-2 distribution [43] rather than an exponential distribution. The autocorrelation function shows a rapid decay with small negative flanks (half-width at half maximum: 10.1 ± 1.1 ms, minimum: 64.6 ± 69.9 ms, mean ± SD).

Without further tuning of network parameters beyond their derivation from slice-physiological and anatomical data, all these *in vivo* statistics are well reproduced by the model (Fig 3). Two-sample Kolmogorov-Smirnov tests did not find notable differences between experimental and simulated distributions in any of the statistics (C_*V*_ : p = 0.26, KS(29) = 0.28; mean ISI: p = 0.4, KS(29) = 0.23; CC: p = 0.4, KS(29) = 0.24), indicating that simulated distributions were not statistically distinguishable from the experimental ones. The asynchronous- irregular firing with low rates is also seen in the raster plot of spike times (Fig 3B).

**Low fraction of spiking neurons and layer-dependent firing rates.** Fig 3B reveals a relatively low fraction of spiking pyramidal cells in both layers—only 22% of the cells emitted more than 10 spikes during the 30s of simulated time, which will be used as the definition of “spiking neurons” throughout the paper, in line with [44–46]. Comparing the neural and synaptic parameters of those neurons which fire at a sufficiently high rate (> 0.33 Hz) and those which do not (≤ 0.33 Hz), we find that only the rheobase (and the cell parameters that contribute to it) differs between the two populations: Spiking neurons have rheobases at the lower end of the distribution (42.9 ± 2.1 pA), compared to 69.0 ± 1.6 pA for non-spiking neurons (mean ± SEM; p = 3.5 ⋅ 10^−20^, t(997) = 9.4, two-sided t-test), some of them even firing spontaneously (called “generator neurons” [47]).

While neurons firing at very low rates may go undetected using extracellular single-unit recordings, recording techniques that are less biased toward spiking neurons, such as calcium imaging or *in vivo* patch-clamp, often reveal a large fraction of neurons that are mostly silent (“dark matter theory” of neuroscience, [44–46]). Consistent with these results, the fraction of neurons with more than 10 spikes rarely exceeded 40% in simulations with *in vivo*-like firing patterns (see section “Admissible and realistic range of input currents” below). This can be explained by the way the neurons are activated: While most neurons receive a background current above their rheobase, the high firing rates of the interneurons (Fig 3B) lead to an average membrane potential in the pyramidal cells below the firing threshold (mean difference: -17.3mV, range: -37.2 to -2.2mV for the example shown in Fig 3) that is occasionally kicked above threshold by random fluctuations. This means that the firing rate is mostly determined by the amplitude of the fluctuations of the membrane potential (see below for statistics). These results are qualitatively conserved across the range of input currents for which the overlap between experimental and simulated distributions is reasonably high.

**Fig 4:**
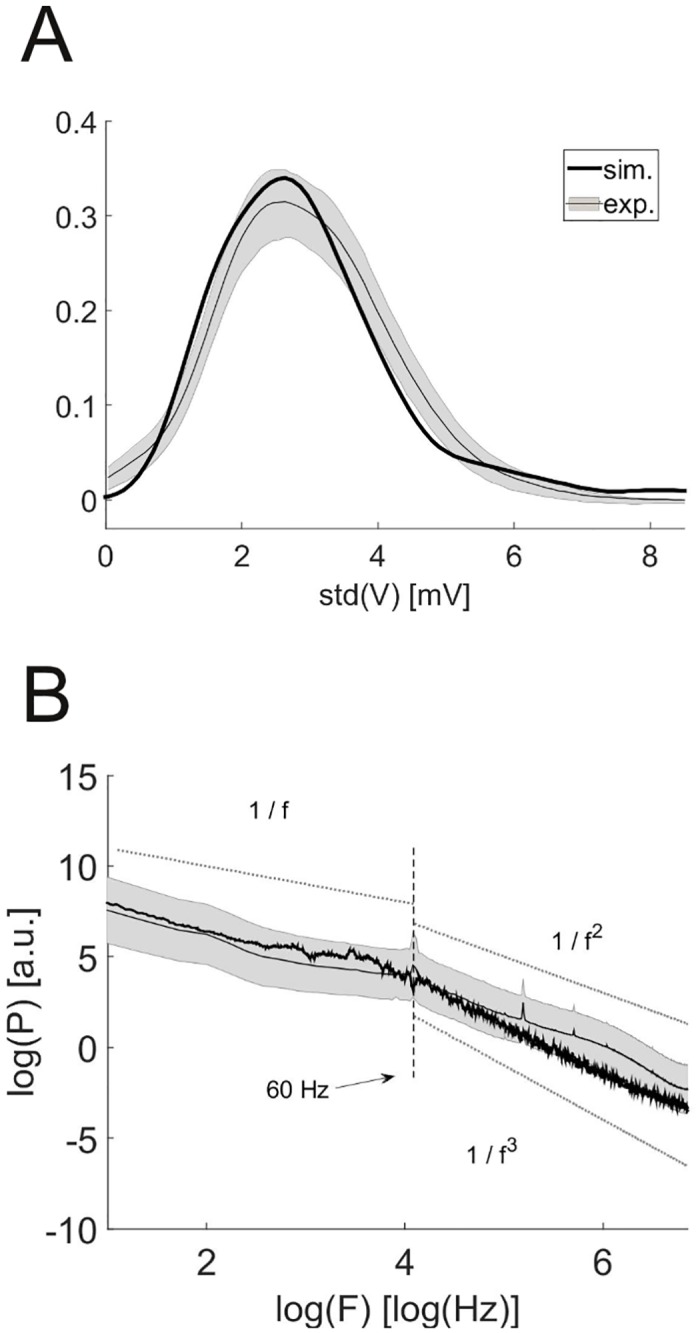
Comparison of simulated membrane and local field potentials with experiments. (A) Estimated distribution of the standard deviation of the membrane potential from anaesthetized rats (gray) and simulated neurons (black) with non-zero firing rates. (B) Power spectrum of the local field potential obtained from experiments (gray) and simulations (black). The dotted lines illustrate the three power laws. The shaded region represents the mean ± the SEM at each point of the experimental distribution, as in Fig 3.

**Membrane potential and local field potential statistics.** In addition to the spike data, we also compared the membrane potential statistics and LFP signals between simulation and experiments. For the simulated network, we observed a broad range of membrane potential fluctuations (after removing spike events; Fig 4A; 3.28 ± 0.72 mV, mean ± SD; range between 0.72 mV and 11.23 mV). We compared this distribution of standard deviations with those from *in vivo* patch-clamp recordings from 10 putative pyramidal cells during up-states in anesthetized adult rodent PFC (kindly provided by Dr. Thomas Hahn, Central Institute of Mental Health and BCCN Heidelberg-Mannheim). The simulated distribution is less than one SEM away from the average of the experimental distribution (pooled over all data sets) for most bins, and a Kolmogorov-Smirnov test (see Materials and Methods) does not show a significant difference (p = 0.45, KS(29) = 0.23). The range of membrane potential fluctuations in the model and in the recordings used here is also consistent with values found in the literature [48, 49].

The local field potential (LFP) in the model was estimated as the sum of all synaptic currents (allowing excitatory and inhibitory currents to partially cancel). This is a reasonable approximation to the standard model of the LFP [50] under the assumption that all neurons are confined in a small volume of cortical space. We computed the power spectral density of this model-derived signal and of the LFP signals obtained from the *in vivo* recordings (Fig 4B). Up to a constant offset (that has been removed in the figure), the spectrum of the simulated LFP is less than one SEM away from the average estimated from the experimental recordings (from awake, behaving animals, also provided by Dr. Christopher Lapish [40]) at most of the frequencies. Both spectra follow a 1/*f* power law for frequencies below 60 Hz and change their scaling behavior for higher frequencies, consistent with LFP spectra described in the literature [51–53] (the fluctuations in the simulated curve are stochastic in nature, i.e. there is no systematic deviation from the 1/*f* behavior across different simulations). For frequencies beyond 60 Hz, the experimental spectrum is well described by a 1/*f*^2^ power law, while the simulated one rather follows a 1/*f*^3^ relation. Both scaling behaviors have been reported in the literature (1/*f*^2^ [52, 54], 1/*f*^3^ [51]), and the difference may result from the simplifications made in the computation of the simulated LFP, e.g. neglecting the spatial integration of currents in extracellular space or the contribution of active currents [14].

**Fig 5:**
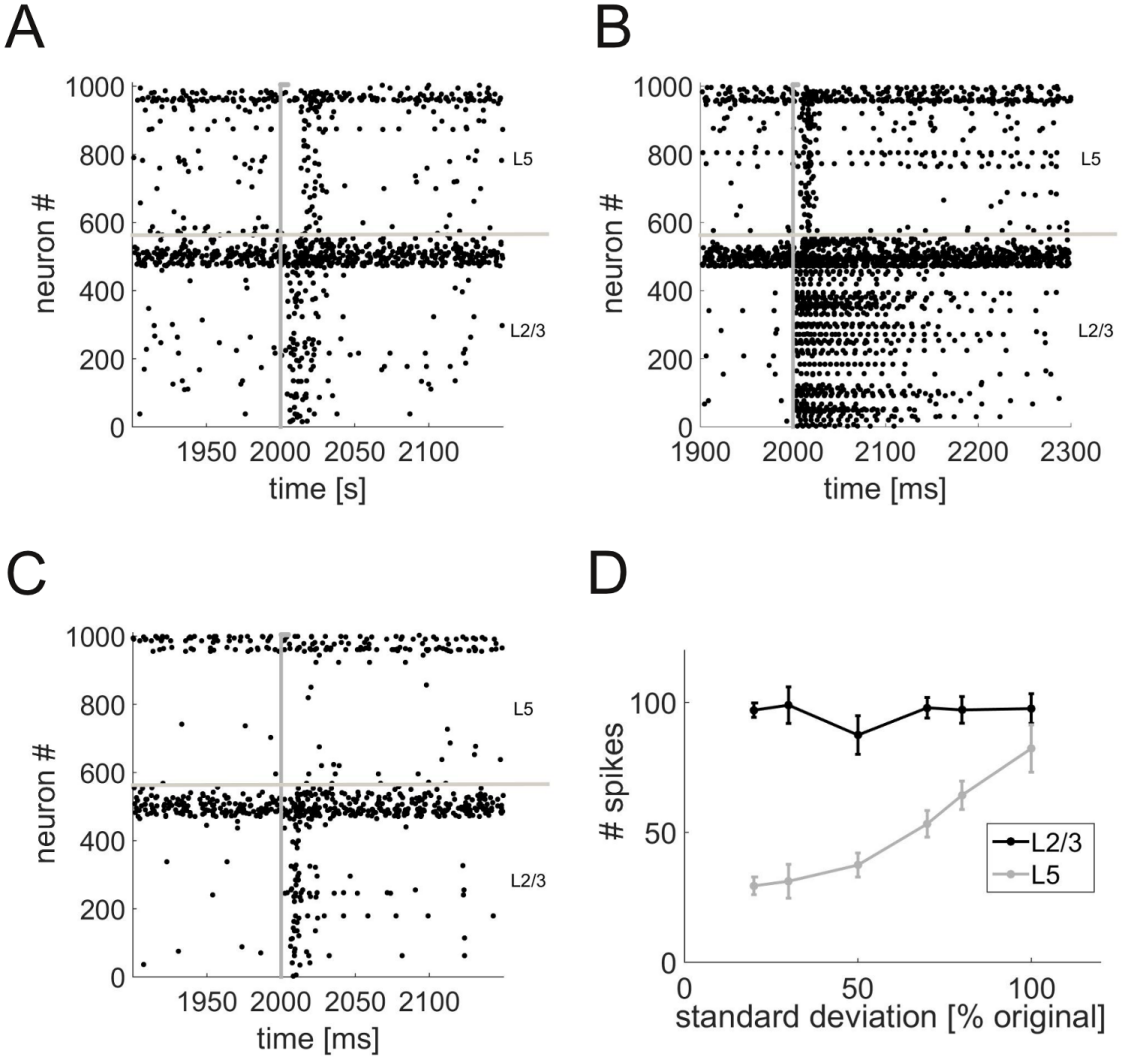
Propagation of transient input. (A) Raster plot of the spike times in the network in response to an external input (gray line) to 10% of the L2/3 pyramidal cells. The input currents are *I*_ex_ = 250 pA, *I*_inh_ = 200 pA. (B) Same as Panel A, but with a stronger (higher rate) external stimulus (see text for details). (C) Same as Panel A, but with neuron parameter variability reduced by 80% (standard deviation set to 20% of its original value). (D) Number of spikes in response to the input as a function of neuron parameter variability. Each data point is the mean ± SEM over a number of repetitions.

**Transient information transfer and the role of neuronal heterogeneity.** We next examined how neurons in L2/3 and L5 would respond to a simple stimulus simulated by a brief series of spikes at high rate (250 spikes within 5 ms) from a virtual (not explicitly simulated) “input population” connected to 10% of the pyramidal cells in L2/3 (cf. Table 3). The stimulus induces a number of spikes in L2/3, and with a short delay also in L5 (Fig 5A). The delays (L2/3: 8.9 ± 1.1 ms; L5: 17.7 ± 1.2 ms, mean ± SD) are similar to values that have been reported in the literature (e.g. 3.4 ± 0.5 ms in L2/3 and 16.6 ± 1.2 ms in L5 [55]). Note that these delays are significantly longer than the fixed synaptic delays (below 2 ms, see Materials and Methods) and arise from the dynamics of the neurons and the kinetics of the synapses (c.f. [56]). For a sufficiently strong stimulus (e.g. 500 spikes within 5 ms), the neurons in L2/3 show a brief period (100–150 ms) of persistent activity (Fig 5B).

The transmission of transient stimuli between layers crucially depends on the heterogeneity of the neuronal parameters. With a 80% reduction in the variance of all parameter distributions (but no change in the means), the stimulus only elicits a response in L2/3, but is not transmitted to the output layer L5 anymore (Fig 5C). Indeed, L2/3 activity is almost independent of neuronal variability, whereas the number of spikes in L5 systematically decreases as the standard deviation of neuronal parameters is reduced (Fig 5D).

To further examine the transmission dynamics, we reproduced an *in vitro* experiment with suppressed inhibition [57] which showed that input in L2/3 resulted in an epileptiform spread of activation across the whole network under this condition, whereas the same input in L5 did not. We mimicked this setup by reducing the inhibitory synaptic weights in the network to 30% of their original values and inducing a strong stimulus (see above) in each of the two layers, while varying the peak conductance *g*_max_ of the synaptic connection between the mimicked Poisson input population and the network. For moderate connection strengths (*g*_max_ = 2), only a fraction of the network responds, and the number of spikes elicited by the network is much larger if the stimulus is injected in L2/3 (404 ± 116, mean ± SD) compared to a stimulus in L5 (118 ± 33). Higher connection strengths (*g*_max_ = 20) reliably drive the network into an “epileptic state” (transient high-rate response from all neurons in the network) for a stimulus in L2/3. In contrast, this state was never reached for an input in L5, consistent with the experimental results in [57].

### Conditions for *in vivo*-like dynamics

In the previous section we showed that the model can reproduce a wide range of characteristics of neural activity *in vivo*. Here, we assess how the reproduction quality of *in vivo*-like behavior depends on those parameters of the model which were only loosely constrained by experimental data. We restrict this analysis to the spike series statistics 〈*ISI*〉, C_*V*_ and CC(0).

**Admissible and realistic range of input currents.** The background currents $I=[I_{\text{ex}}^{\text{L23}},I_{\text{ex}}^{\text{L5}},I_{\text{inh}}^{\text{L23}},I_{\text{inh}}^{\text{L5}}]$ have so far been treated as free parameters, as such estimates are difficult to obtain or at least have not been reported experimentally. We address this in two ways: First, we systematically vary these four currents and assess the similarity between experimental and simulated spike time distributions using Kolmogorov-Smirnov statistics as before. Second, we estimate the required background currents from the simulation itself, using the assumption that the simulated network is embedded in a larger, but structurally identical network from which these currents originate.

**Fig 6:**
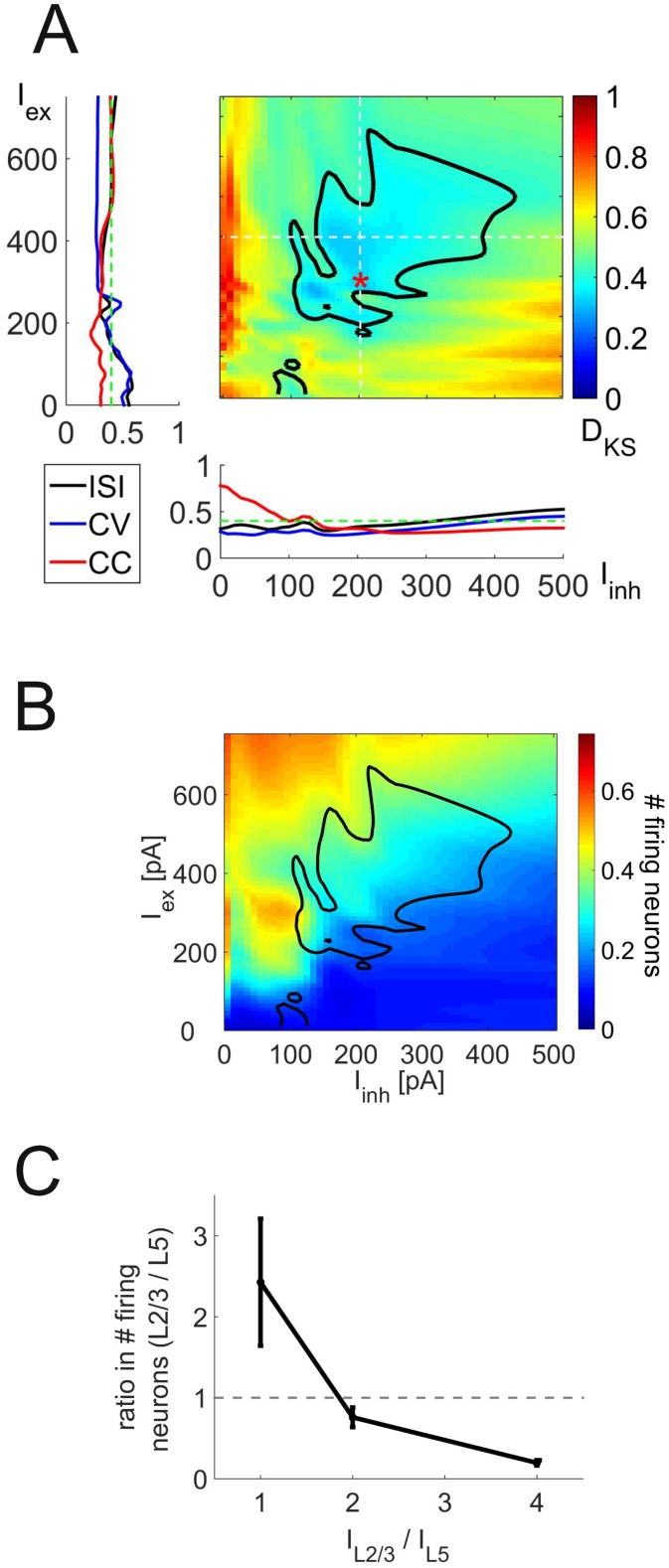
Dependence of network behavior on the magnitude of synaptic background inputs. (A) Maximum of the Kolmogorov-Smirnov test statistic (*D*_KS_) comparing the experimental and respective simulated distributions for the mean ISI, C_*V*_, and cross-correlation as a function of input currents into excitatory (*I*_ex_) and inhibitory (*I*_inh_) neurons in layer 2/3. *D*_KS_ values within the blackly delineated area have *p* values larger than 0.05 for each of the three tests. The insets show the three individual *D*_KS_ values as a function of one of these input currents alone (for *I*_inh_ = 200 pA in the left and *I*_ex_ = 400 pA in the lower inset, indicated by the white dotted lines). *D*_KS_ values above 0.4 (green lines) correspond to significant (*p* = 0.05) deviations from experiments in the given distribution. The red asterisk indicates the parameter set used for the simulations presented in the previous figures. (B) Fraction of neurons emitting at least 10 spikes during a 30 sec simulation period for the same currents used in Panel A. The blackly delineated area was copied from Panel A and superimposed on the current graph. (C) Ratio of the number of spiking pyramidal cells between layers 5 and 2/3 as a function of the input current ratio into pyramidal cells in layers 2/3 and 5. Each data point represents the mean ± SEM over three different ratios of input currents into interneurons in layers 2/3 and 5 and a number of $I_{\text{ex}}^{\text{L23}}$ and $I_{\text{inh}}^{\text{L23}}$ values.

Fig 6A shows the Kolmorgorv-Smirnov test statistic *D*_KS_ as a function of *I*_ex_ and *I*_inh_, where $I_{\text{ex}}^{\text{L23}}=I_{\text{ex}}^{\text{L5}}=I_{\text{ex}}$ and $I_{\text{inh}}^{\text{L23}}=I_{\text{inh}}^{\text{L5}}=I_{\text{inh}}$ (see below for a discussion of laminar differences in the input currents). The figure reveals that the overlap between experimental and simulated distributions is acceptable (*p* > 0.05 for the two-sample Kolmogorov-Smirnov test, i.e. failure to reject the null hypothesis *H*_0_ of equal distributions for the two samples, see Materials and Methods) for a wide region of *I*_ex_ and *I*_inh_ values (delimited by the black isocline in Fig 6A, associated with *D*_KS_ values below 0.4). More specifically, simulated C_*V*_ and mean ISI distributions become indistinguishable from their experimental counterparts as *I*_ex_ increases, while the overlap with the ISI distribution decreases again for very high *I*_ex_ values (Fig 6A, left inset). Both C_*V*_ and mean ISI deviate from the experimental distributions as *I*_inh_ increases. CC, on the other hand, matches well with the experiments for high *I*_inh_ values (Fig 6A, lower inset). As mentioned above, the fraction of firing neurons is quite low in most networks showing *in vivo*-like firing patterns, typically between 20 and 30%, as shown in Fig 6B (blackly delimited region gives the empirically acceptable parameter regime copied from Fig 6A).

The ratio of inputs into the two layers, $I_{\text{ex}}^{\text{L23}}/I_{\text{ex}}^{\text{L5}}$ and $I_{\text{inh}}^{\text{L23}}/I_{\text{inh}}^{\text{L5}}$, does not have a strong influence on these results within the tested range (mean *D*_KS_ ± SEM: 0.31 ± 0.05, 0.32 ± 0.05 and 0.35 ± 0.06 for ratios of 1, 2 and 4, respectively), but does of course affect the relative firing rate between the two layers. *In vivo* experiments found that firing rates are considerably higher in L5 compared to L2/3 pyramidal cells (3–20 times [27]). This condition is fulfilled in our model as long as L2/3 receives less or the same input as L5 (Fig 6C).

To estimate which range of *I* values could be realistically assumed, we tested whether a substantially larger network than the 1000-neuron-network simulated here would produce mean synaptic currents that are large enough to self-sustain *in vivo*-like activity (i.e. within the blackly circumscribed regions in Fig 6A). In this case, the activity in the large network and the small network (the latter driven by the currents from the larger one) would be indistinguishable, and the *in vivo*-like activity would be supported by the larger network. We increased the size of the network either by changing the density of neurons or by adding input from nearby columns (see “Estimation of background currents” in Materials and Methods).

**Fig 7:**
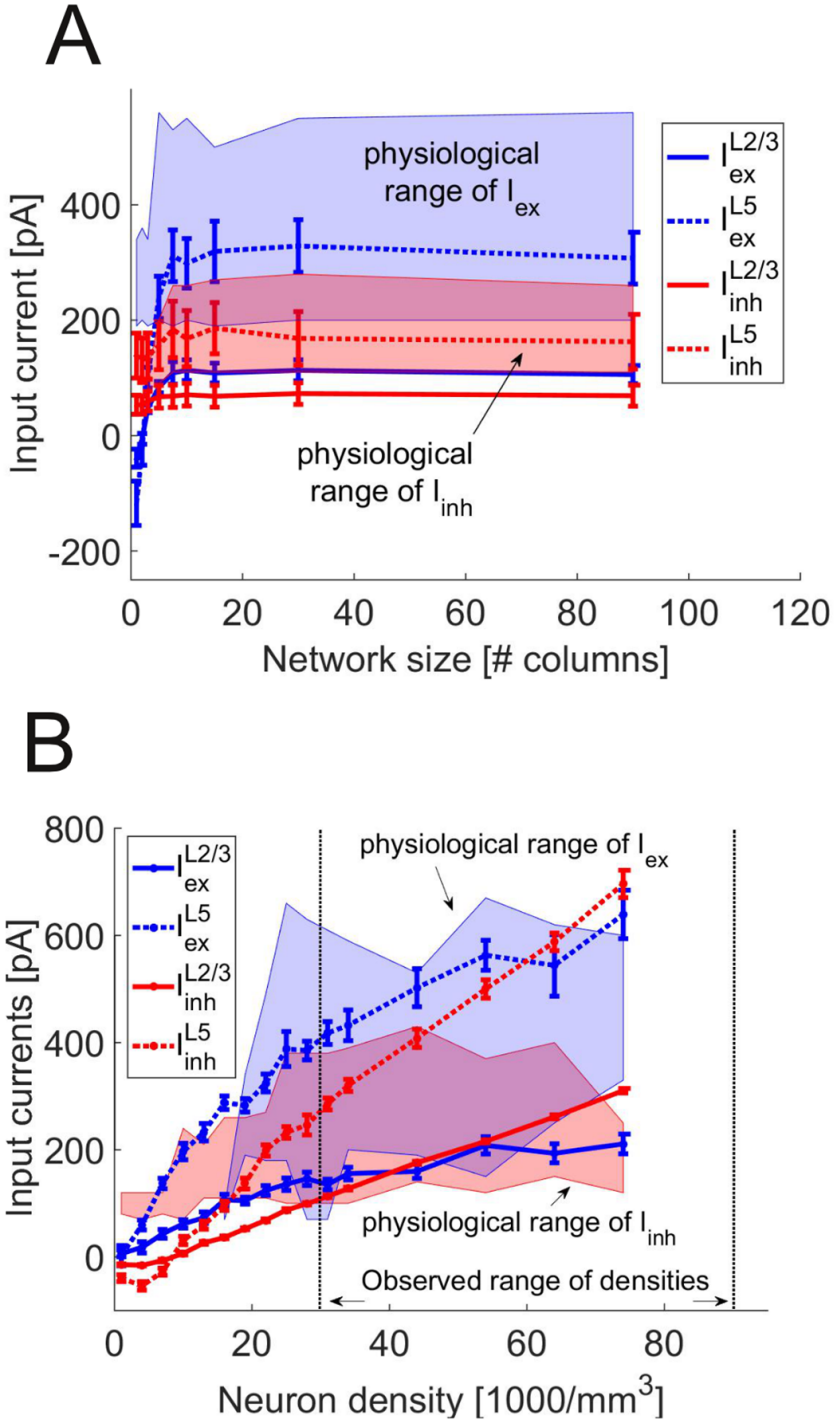
Scaling of total synaptic input current with network size. (A) Synaptic input current as a function of the number of columns. Shown are the averaged values over different neuron densities (mean ± SEM) as a function of column number for the inputs into L2/3 pyramidal cells (solid blue), L2/3 interneurons (solid red), L5 pyramidal cells (dotted blue) and L5 interneurons (dotted red). The region of currents which yield *in vivo*-like behavior (cf. black region in Fig 6A, *D*_KS_ < 0.4) is marked in blue for *I*_ex_ and in red for *I*_inh_. (B) Same as in A, but synaptic input as a function of total cell density, averaged over column numbers ≥ 5. The dotted horizontal lines show the upper and lower bound of densities found in anatomical studies.

Fig 7 shows the mean synaptic current into pyramidal cells and interneurons in L2/3 and L5 that would result from the reduced equivalent-population input models described in Materials and Methods if the network size was varied through the number of columns (Fig 7A) or the density of neurons within columns (Fig 7B, both figures showing currents averaged over the values of the other independent variable, i.e. neural density or number of columns, respectively). The shaded areas show the ranges for *I*_ex_ (blue) and *I*_inh_ (red) within which these currents would produce *in vivo*-like activity (*D*_KS_ < 0.4). Note that it is sufficient that one of the two layers receives a current above the lower bound, as it will push the other layer into the right regime by cross-layer synaptic connections. The upper bound, on the other hand, may not be exceeded by either of the two layers, as this would push the other layer beyond its upper bound as well. It is apparent that these conditions are fulfilled already for (spatially) relatively small networks (∼ 5 columns), and currents saturate as network size grows further (Fig 7A). By increasing the neuron density, on the other hand, the input currents increase monotonically over a wide range (Fig 7B, averaged over all column numbers ≥ 5). Mean synaptic currents sufficient to drive the network into the experimentally observed regime arise for densities between 19,000 and 44,000 neurons per mm^3^. This range overlaps with densities found in anatomical studies (30,000 to 90,000 neurons per mm^3^ [58–60]; horizontal dotted lines).

**Variation of synaptic parameters.** We attempted to estimate all synaptic parameters from data reported in the literature. Given that these come with some uncertainty and variation, however, we explored how sensitive the network behavior is with respect to changes in mean synaptic peak conductances and their distribution, synaptic time constants, and the GABA_A_ reversal potential. All these parameter variations were performed for a range of different background currents and averaged results are reported.

**Fig 8:**
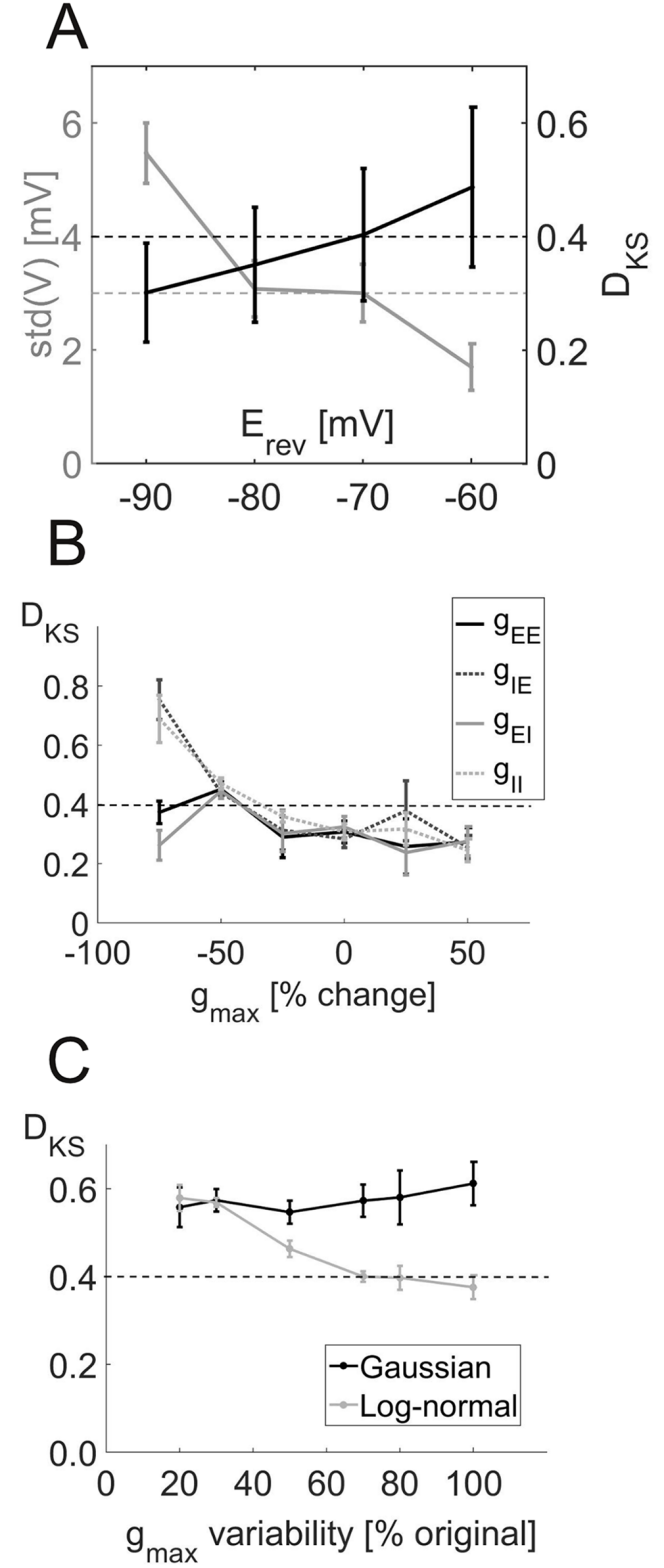
Effect of synaptic parameter changes on network activity. (A) Maximum of the Kolmorov-Smirnov test statistics (*D*_KS_) comparing the three experimental and simulated distributions (black) and standard deviation of the simulated membrane potential (gray) for different GABA_A_ reversal potentials. Each data point is the mean ± SEM over several values of input currents. The black line denotes the *D*_KS_ limit of 0.4 above which differences become significant (p ≤ 0.05), and the gray line marks the average of the experimentally observed standard deviations (cf. Fig 4A). (B) *D*_KS_ values as a function of percent change in overall synaptic peak conductances between pyramidal cells (E) and interneurons (I). The dotted line denotes the critical *D*_KS_ value of 0.4 (see above). (C) *D*_KS_ values for different values of the standard deviation of the synaptic peak conductances using either the original log-normal distribution (gray curve) or a Gaussian distribution with the same mean and standard deviation (black curve). As above, the dotted line marks the critical *D*_KS_ value of 0.4. In all figures, each data point shows the mean ± SEM over the *D*_KS_ values for a number of different input currents.

The GABA_A_ reversal potential $E_{\text{rev}}^{\text{GABA}}$ was initially set to -70mV, which is well within the range of the values reported in the literature [19, 24, 55, 61]. Within the physiologically reasonable range from -90 to -60mV [62], the divergence between simulated and experimental distributions (as assessed by the KS test statistic) increases with $E_{\text{rev}}^{\text{GABA}}$ (Fig 8A). At the same time, the standard deviation of the membrane potential decreases. The time constants of the synaptic kinetics also turned out to be important for the agreement with *in vivo* data: While small changes are acceptable, both very fast and very slow GABA_A_ kinetics strongly diminish the agreement with the experimental data (*D*_KS_ = 0.99 for *τ*_on_ = 0.6 ms and *τ*_off_ = 8 ms and *D*_KS_ = 0.85 for *τ*_on_ = 12 ms and *τ*_off_ = 160 ms). The NMDA time constants have less effect, unless they are very strongly increased (*D*_KS_ = 1.0 for *τ*_on_ = 17.2 ms and *τ*_off_ = 300 ms, compared to values of *τ*_on_ ≤7 ms and *τ*_off_ ≤ 100 ms reported in the literature [38, 39]). The effects of the mean synaptic peak conductances are shown in Fig 8B. While small to moderate changes ( ± 50%) have no significant effect, a strong decrease in the inhibitory synaptic efficiencies leads to a significant mismatch with the *in vivo* statistics.

Apart from the mean, we also analyzed how the distribution of the synaptic peak conductances affected *in vivo*-like behavior by either reducing the variability or drawing them from a normal rather than a log-normal distribution (conserving mean and standard deviation). Reducing the variability of the synaptic weights increased the mismatch between empirical and simulated distributions (Fig 8C, gray line). Surprisingly, just changing the form of the underlying distribution from log-normal to normal, without changing its mean or standard deviation, had a similarly strong effect as a pronounced reduction in standard deviation (Fig 8C, black line), so both the variability as well as the functional form of the synaptic conductance distribution are crucial for reproducing spiking dynamics as observed *in vivo*. Without the long tail of the log-normal distribution, the network activity becomes much more synchronized (CC(0): 0.017 ± 0.006, mean ± SD) and exhibits strong bursts (C_*V*_ : 1.81 ± 0.09, mean ± SD), while mean firing rates are not much affected (〈ISI〉: 420 ± 558 ms, mean ± SD).

## Discussion

We presented a model of the prefrontal cortex which is entirely defined by electrophysiological and anatomical data, and is capable of reproducing a wide range of *in vivo* statistics, including properties of single spike trains and pairwise correlations, the power spectrum of the local field potential and the variability of the membrane potential. Importantly, this reproduction did not require specific tuning of model parameters towards *in vivo* behavior. In fact, variation of the synaptic parameters shows that the ability of the network to show *in vivo*-like behavior is robust against considerable changes. In particular, it is possible to increase or decrease the synaptic weights by up to 50% of their original value without significant changes in the prediction performance (Fig 8B). This keeps the model flexible to synaptic plasticity, i.e. the weights can be modified by task-related learning rules without changing the global activity of the network. The only variable that was not tightly constrained by *in vitro* data, the external input currents, showed a wide range of admissible values, within a range that would be produced by a network supposedly large enough to self-sustain activity. Despite the high biological validity, the model remains simple enough to allow for efficient simulation, due to the combination of a simple, but versatile neuron model and a complex network structure. To our knowledge, no other biophysical model currently exists that is as tightly constrained by the specific neuronal and synaptic properties of the prefrontal cortex and systematically compared to *in vivo* data as the one presented here.

### Relation to other network models

The current model has a strong focus on its tight connection to data. Many existing network models of the neocortex are based on neurobiological findings as well [13, 14, 63–65], but the present model differs from them in two respects: The strict way in which the *in vitro* data is used to fix or systematically infer every detail of the model, and, more importantly, the quantitative test of the model’s validity on a wide range of *in vivo* findings. Recently, a few studies have also moved in this direction. Fisher et al. [66] proposed a model for the short-term memory circuit in the oculo-motor system of the adult goldfish. They fitted the model simultaneously to a range of anatomical, physiological and behavioral data. This approach gives a coherent picture of this particularly well-defined non-cortical system. Furthermore, Potjans and Diesmann [27] proposed a model of a sensory cortex network where the connection probabilities are thoroughly derived from in vitro studies. While the neuron parameters are generic and homogeneous, their focus is on the precise laminar and horizontal organization of the synaptic connections. They compare the results of their simulations with the baseline firing rates and flow of transient information through the different layers *in vivo*. These comparisons to experimental data are qualitative in character, as it is the case for most existing large-scale models of cortical networks [12–14, 67]. However, a few recent studies also made statistical comparisons on partial aspects of physiological data [68, 69]. It would be interesting to assess these models on a wider range of *in vivo* data as we proposed here, to see which degree of biological detail is sufficient to predict their key properties.

An important simplification made in the present model is the reduction to two laminar components, leaving out layer 4 and 6 as well as the long-range fiber bundles and interneurons in layer 1. While layer 4 is missing in rodent PFC, layer 6 is only weakly connected to the other layers in our reference connectivity maps, which are based on the motor cortex [57, 70, 71]. Thus, its inclusion in the network should not have a major impact on the results shown here. This is probably different in sensory networks, where layer 6 strongly interacts with both pyramidal cells and interneurons in layer 4 [72].

### The relevance of synaptic and cellular heterogeneity

The model exhibits a low fraction of spiking neurons, consistent with results from recording methods such as calcium imaging, which are not biased towards high firing rates (“dark matter theory” of neuroscience [44–46]). As described above, this may partly result from the variance-driven firing of the neurons: The membrane potential is on average well below the spiking threshold, but large fluctuations still lead to occasional spiking. The size of the fluctuations and the low-rate, Poisson-like firing (C_*V*_ ≈ 1) of the neurons is consistent with the high-conductance state theory [48] and balanced-state theory [42, 73]. We note that the irregular and highly asynchronous firing of the neurons [74] observed here is a generic property of the network that simply emerged from its parametrization through *in vitro* and anatomical findings.

There are two main determinants of the high-fluctuation regime of the model: First, variability in the membrane potential requires variability in the synaptic parameters and in particular, the fat tail of the log-normal distribution of the synaptic weights. Second, the range between the firing threshold *V*_up_ and the GABA_A_ reversal potential $E_{\text{rev}}^{\text{GABA}}$ must be sufficiently large, because below $E_{\text{rev}}^{\text{GABA}}$, all synaptic currents depolarize the cell, so the dynamical range for a balanced, variance-driven state is constrained between these two values.

Using the multivariate distributions of neuron parameters obtained from our *in vitro* recordings, we also observed that decreased cellular heterogeneity has a profound effect on the processing of transient stimuli. It prevents the transmission of stimulus-induced activity from L2/3 to L5. This phenomenon can be understood if one considers the rheobase distribution: Reduced heterogeneity removes those neurons that originally had a very low or even negative rheobase. These are the ones which are highly susceptible to even small inputs and form a small but significant fraction of L5 neurons that were activated by the transient synaptic input from the L2/3 cells. Given that L5 provides the majority of output to other brain areas, impaired transfer of stimuli to this layer may lead to major impairments in information processing.

Thus, apparently quite subtle changes in the distributional properties of synaptic and cellular parameters (not affecting their means) may lead to major changes in network dynamics and functional connectivity among columns or areas, effects that have been proposed to underlie major psychiatric conditions like schizophrenia [8, 9].

### Self-sustained activity of the PFC

By varying the total input from a virtual population designed according to the same principles as the actually simulated network, we provided evidence that a larger network than the one actually simulated with anatomically realistic neuron densities should be capable of self-sustaining *in vivo*-like spiking modes. Although we did not demonstrate self-consistency in a strict sense, we have shown that the background currents into the smaller, simulated network needed to yield *in vivo*-like behavior are consistent with the range produced by a much larger network of anatomically reasonable size. For currents within the blackly delimited region of Fig 6A, the spike train distributions are statistically indistinguishable from the *in vivo* statistics, and the background currents that would result from scaling up the simulated network to anatomically realistic size lie exactly within this regime. This analysis implies that *in vivo*-like activity can be self-sustained in a larger network with the same anatomical layout as explicitly simulated here, as it has been observed for instance in deafferented cortical slabs [75], while e.g. the thalamus or other sub-cortical structures may provide transient, stimulus-related input or modulate the overall activity of the network [76].

Interestingly, the currents produced by this procedure are much higher in L5 compared to L2/3 (Fig 7), as required for the much higher firing rates observed *in vivo* [27]. At first glance, this seems counterintuitive, as L2/3 neurons receive input from neighboring columns, while L5 neurons do not (see “Estimation of background currents” in Materials and Methods). However, L5 also receives strong inputs from L2/3, while the inverse projections are much weaker (Fig 2A). Thus, once L2/3 neurons receive enough input from other columns to spike, they drive L5 much stronger than themselves.

In terms of space, input from just a few columns is sufficient to drive the network, as connectivity rapidly decays over the cortical extent. Nevertheless, a single column is not sufficient for driving the network because of the higher fraction of excitatory synapses in long-range connections and the more local connectivity of interneurons. This is consistent with recent experimental studies [60, 77] and earlier results from deafferented cortical slabs [75] (but see [78]).

### Possible applications

In this study, we have focused on the resting state of the network. However, it may also be used as a foundation for more functional investigations of cognition. For instance, the clusters of increased synaptic connectivity may serve as building blocks for cell assemblies [29] which can be used to represent behavioral rules [3, 40] or transient stimuli that need to be kept in working memory [64]. Moreover, the fast and fully automatized framework for fitting the neuron model to *in vitro* data [15] opens a convenient way to test the network effects of genetic or pharmacological manipulations: Recordings from neurons that underwent such a manipulation can be used by the very same fitting procedure as employed for wildtype or control cells, resulting in different parameter sets that can be plugged into the network to assess their implications for network behavior. Likewise, this could be done for the synaptic parameters using paired recordings and recent methods to fit the parameters of short-term synaptic plasticity models to these data [79].

In summary, we have provided a prefrontal cortex network model here with single cells and synapses strictly parametrized through in vitro electrophysiological findings (no specific tuning or adjustment of synaptic currents to compensate for simulated network size), with realistic cellular and synaptic heterogeneity, and with a structural layout derived from anatomical data. We have then systematically compared the full network activity to a number of spiking and correlation statistics from *in vivo* multiple single cell recordings in awake rodents, as well as LFP data from these animals, and estimates of membrane potential fluctuations from in vivo patch-clamping. Our model is therefore highly validated at the *in vivo* physiological level, yet it is computationally efficient by virtue of its computationally comparatively simple single unit design. We therefore hope that this network model can serve as a valuable tool in the further study of how physiological and anatomical properties relate to cortical network dynamics, and ultimately cognition, and how alterations of these properties may give rise to symptoms observed in various psychiatric conditions.

## Materials and Methods

### Model specification

**Neuron model.** Single neurons were modeled by the simplified adaptive exponential integrate-and-fire neuron (simpAdEx) introduced in [15]:

$$C\cdot \frac{dV}{dt}=-g_{L}\cdot \left(V-E_{L}\right)+g_{L}\cdot \Delta_{T}\cdot e^{\left(\frac{V-V_{T}}{\Delta_{T}}\right)}+I-w=:w_{V}-w$$ (1)

$$\frac{dw}{dt}=\left\{\begin{array}{cc} 0 & \;\;\;\text{for}\;\left|w-w_{V}\right|>\frac{\tau_{m}}{\tau_{w}}w_{V}\\ \Theta \left(V_{T}-V\right)\cdot [1-\frac{\tau_{m}}{\tau_{w}}]\frac{dw_{V}}{dV}\frac{dV}{dt} & \;\;\;\text{otherwise} \end{array}\right.$$ (2)

$$\text{if}\;V>V_{\text{up}}\;\text{then}\;V\to V_{r}\;\text{and}\;w\to w_{r}=w+b$$

$$\text{if}\;w=\left(1+\frac{\tau_{m}}{\tau_{w}}\right)w_{V}\;\text{then}\;w\to \left(1-\frac{\tau_{m}}{\tau_{w}}\right)w_{V},$$

where C is the membrane capacitance, *g*_*L*_ a leak conductance (with reversal potential *E*_*L*_), *τ*_*m*_ and *τ*_*w*_ are the membrane and adaptation time constants, respectively, *Θ* denotes the heavy-side function, and *w*_*V*_ is the V-nullcline of the system as defined in Eq 1. Like the full AdEx [80], this model consists of one differential equation for the membrane potential *V* (including an exponential term with slope parameter Δ_*T*_, which causes a strong upswing of the membrane potential once it exceeds *V*_*T*_), and one for an adaptation variable *w*, and can reproduce a whole variety of different spiking patterns [15]. A spike is recorded whenever V crosses *V*_up_, at which point the voltage is reset to *V*_*r*_ and spike-triggered adaptation is simulated by increasing *w* by a fixed amount *b*. The simpAdEx was derived from the full AdEx based on phase-plane considerations, effectively dissecting the dynamics into three different regimes (defined through their distance from the V-nullcline, see Eq 2), each of them approximated in a way that allows for closed-form expressions for the instantaneous and steady-state firing rates. This enables fast and efficient fitting of the model to f-I and I-V curves as commonly used to characterize the electrophysiological behavior of cells *in vitro* [15] (Fig 1A).

We had shown previously that this model, although estimated from f-I and I-V curves only, can predict spike times under *in vivo*-like conditions with high accuracy from physiological recordings not used for model fitting [15]. Different from [15], the upper voltage limit *V*_up_ was initially estimated from the inflection point of the voltage traces. This makes *V*_up_ an absolute firing threshold (as in the leaky integrate-and-fire neuron) and leaves *V*_th_ as a free parameter for the subthreshold dynamics, resulting in a shallower exponential rise to the spike, more akin to what would be expected from the action of persistent sodium channels [81] or L-type calcium channels [82].

We estimated neuron models for a large number of *in vitro* recordings from different cell types from the prefrontal cortex of rats and mice, namely layer 3 (n = 34) and layer 5 pyramidal cells (n = 108), fast-spiking (n = 32), and bitufted (n = 22) interneurons. Additionally, we extracted statistics (means and variances) about f-I curves and subthreshold dynamics of Martinotti cells from the literature [26, 83–88], and used these to construct 100 sets of f-I and I-V curves drawn from Gaussian distributions instantiated by the empirically estimated parameters. For each data set drawn from these distributions, Martinotti cell models were estimated. The pool of estimated models for each cell type defines a multivariate parameter distribution for each type of neuron, from which the final parameter sets for the 1000 neurons used in the network simulations were drawn. This joint parameter distribution for each cell type was initially modeled as a multivariate Gaussian, where marginal distributions not of Gaussian shape (as estimated from the empirical data) were first Box-Cox-transformed to adhere with the Gaussian assumptions. In a second step, the Box-Cox transform was inverted to regain the non-Gaussian shape of the marginal distributions (red curves in Fig 1C). The mean values and standard deviations of all model parameters for the different cell types are given in Table 1.

**Network anatomy and connectivity.** The network is divided into two laminar components, representing the superficial layers L2/3 and the deep layer L5 (Fig 2A). The network also includes a horizontal organization into distinct columns which are typically about 300*μm* wide [89], so the model is in principle suited to study information transfer between columns. For most part, however, the present analyses focuses on a single column which was found to be sufficient to reproduce *in vivo*-like resting-state activity, provided a source of constant external input (see below). The relative numbers of pyramidal cells and interneurons in each layer were taken from [58] who studied the rat motor cortex, as such data are not available for the PFC. Following [58], 47% of all cells were modeled as L2/3 pyramidal cells (L2/3-E), 10.4% as L2/3 interneurons (L2/3-I), 38% as L5 pyramidal cells, and 4.6% as L5 interneurons. With regards to the specific types of interneurons and their distribution across layers, we followed [90] and [91] and defined local interneurons (IN-L) with projections within the same layer and column as fast-spiking cells, cross-layer interneurons (IN-CL) as bitufted cells, and far-reaching interneurons (IN-F) with projections both outside of their column and layer of origin as Martinotti cells [90] (Table 1). The cross-column cells (IN-CC) have been classified as large basket cells [90, 92, 93], with electrophysiological properties resembling those of pyramidal cells [94]. Therefore, we used the same parameter distributions as for the pyramidal cells in the respective layer for this cell class. Markram et al. [90] also estimated the relative numbers of different types of interneurons within each cortical layer. Together with the classification above, these data result in the full distribution of cell types summarized in Table 2.

Neurons were randomly connected with distinct connection probabilities *p*_con_ for each pair of cell types as derived from a survey of about 40 studies, e.g. [95–99], most of which are reviewed in [22] and [27], except [18–21, 23–25] and [26]. Most of them performed whole cell or dual sharp electrode recordings *in vitro* in various neocortical regions of rats and mice. We also included a few studies using monkeys, ferrets and cats, as there are more studies from PFC in these species and some parameters were not available in rodents. Connection probabilities were further adjusted jointly with the connection weights to match data from photostimulation experiments as explained in detail below [57, 70, 71, 100, 101]. Pyramidal cells within the same layer form clusters of increased connection probability as defined by the “common neighbor rule” [28, 29] which states that the connection probability of two neurons increases linearly with the number of neurons they are both connected to. Furthermore, a fraction of 47% of the connections was specified as reciprocal [32], since the proportion of reciprocal connections was experimentally observed to be significantly higher than chance [32, 37]. For cross-column projections, connection probabilities exponentially decay with the distance from the column of origin. Data from rodent studies [29, 60, 77, 102, 103] suggest a wide range of spatial decay constants. We use the median from these studies, which is 114*μm* for pyramidal cells and 95*μm* for interneurons.

Apart from the recurrent synaptic connections within the network, we also introduced *constant* background currents that are fed into all neurons and which differ in strength for pyramidal cells and interneurons in layer 2/3 and 5. Appropriate values for these four streams of background inputs were determined using reduced equivalent-population input models (see below). It is emphasized that there was no source of external noise fed into the network, i.e. the external inputs consisted of just constant (DC) currents. Thus, all variability observed in the network arises from its internal dynamics.

**Synaptic properties.** Neurons were connected through conductance-based AMPA-, GABA_A_-, and NMDA-type synapses, with kinetics modeled by double exponential functions [104]

$$\begin{array}{ll} I_{\text{X}}= & g_{\text{X}}^{\text{max}}\;s(V)\sum_{t_{\text{sp}}}^{}a(t_{\text{sp}})(e^{-(t-t_{\text{sp}}-\tau_{D})/\tau_{\text{off}}^{\text{X}}}-e^{-(t-t_{\text{sp}}-\tau_{D})/\tau_{\text{on}}^{\text{X}}})(V-E_{\text{rev}}^{\text{X}})\\ & \text{with }s(V)=\{\begin{array}{rr} 1.08{\left(1+0.19\cdot \text{exp}(-0.064V)\right)}^{-1} & \text{for X}=\text{NMDA}\\ 1 & \text{otherwise} \end{array} \end{array}$$ (3)

where *X* ∈ {AMPA, GABA_A_, NMDA}. The reversal potential *E*_*rev*_ is set to zero for AMPA and NMDA, and to −70 mV for GABA_A_ [19, 24, 55, 61]. The onset and offset time constants *τ*_on_ and *τ*_off_ are set to 1.4 ms and 10 ms, respectively, for AMPA [39, 94], 3 ms and 40 ms for GABA_A_ and 4.3 ms [105] and 75 ms [38, 39] for NMDA. NMDA conductances exhibit a nonlinear voltage-dependency *s*(*V*) due to their magnesium block at lower voltages [106]. Synaptic transmission delays *τ*_*D*_ were drawn from Gaussian distributions with means and standard deviations depending on the pair of connected cell types, with parameters derived from the same electrophysiological literature as the connection probabilities (see below; Table 3). Synaptic delays were chosen to increase linearly with distance from the target column [107], *τ*_*D*_(*d*) = *τ*_*D*_(1 + *d*), where *d* is the number of columns separating the connected neurons.

Synapses were also equipped with short-term plasticity dynamics implemented by the corrected version [108] of the Tsodyks and Markram model [30]

$$a_{k}=u_{k}\cdot R_{k}$$ (4)

$$u_{k}=U+u_{k-1}\left(1-U\right)\exp\left(-\Delta_{k-1}/\tau_{\text{fac}}\right)$$ (5)

$$R_{k}=1+\left(R_{k-1}-u_{k-1}R_{k-1}-1\right)\exp\left(-\Delta_{k-1}/\tau_{\text{rec}}\right).$$ (6)

These recursive equations describe the dynamics of the relative efficiency *a*(*t*_sp_k__) across series of spikes, with initial conditions *u*_1_ = *U* and *R*_1_ = 1, where *t*_sp_k__ is the interval between the (*k* − 1)th and the *k*th spike. Model parameters *U*, *τ*_rec_ and *τ*_fac_ were specified according to [31] and [32] who differentiated between facilitating (E1/I1), depressing (E2/I2) or combined (E3/I3) short-term dynamics, for both excitatory (E) and inhibitory (I) connections (Fig 2B, right panel; Table 4). The cell types of the pre- and postsynaptic neurons determine which of these classes is used for each individual combination (Fig 2B, left panel). Synaptic inputs were further subject to release failures with a probability of 30% [33–36].

**Table 4:** Short-term synaptic plasticity.

parameter	U	*τ*_rec_ [ms]	*τ*_fac_ [ms]
E1	0.28 (0.02)	194 (18)	507 (37)
E2	0.25 (0.02)	671 (17)	17 (5)
E3	0.29 (0.03)	329 (53)	326 (66)
I1	0.16 (0.10)	45 (21)	376 (253)
I2	0.25 (0.13)	706 (405)	21 (9)
I3	0.32 (0.14)	144 (80)	62 (31)

Mean and standard deviation of the parameters of the six types of short-term synaptic plasticity.

Distributions of peak conductances (“synaptic weights”) *g*_max_ for each cell population were derived in two steps. As the first step, initial estimates were obtained from the anatomical and electrophysiological literature (see above). Generally, peak conductances were adjusted such that they reproduced log-normal distributions of postsynaptic potential (PSP) amplitudes as reported in [37] (means and standard deviations given in Table 3). For excitatory synapses, only the AMPA conductances are specified this way, while NMDA conductances are given by 1.09 times the respective AMPA peak conductance [38, 39], with both AMPA and NMDA synapses activating after the same delay. For synaptic connections where peak conductances were not directly available from the surveyed literature, estimates were obtained in one of the following ways: 1) Missing estimates for specific interneuron types were replaced by estimates from other interneuron types where possible. 2) Missing estimates for inhibitory connections within one layer were replaced by those from another layer, rescaled such that they followed the same between-layer ratio as the excitatory inputs. 3) If only means but no standard deviations for the distribution of synaptic parameters were available, we used standard deviations from another layer scaled according to the ratio of the means between layers (missing values of connection probabilities or synaptic delays were estimated in the same way). Finally, for cross-column projections, synaptic weights were assumed to decay with the same exponential course (space constant of 114*μm* for pyramidal cells and 95*μm* for interneurons) as taken for the connection probabilities themselves (see above).

In a second step, since by far most of the studies cited above have been performed in sensory areas, data from laser scanning photostimulation (LSPS) [57, 70, 100, 101] and genetically targeted photostimulation [71] studies from motor cortex were used to obtain values closer to PFC. Specifically, all connection probabilities and synaptic weights were scaled such that the total input to each cell type would match the one observed experimentally in these studies. To compute this scaling factor $s_{i,j}^{I}$, the product *p*_con_ ⋅ *g*_max_ of connection probability and synaptic weight was assumed to be proportional to the quantity *I*_LSPS_ obtained in the experimental studies [70], yielding

$$s_{i,j}^{I}=\frac{I_{\text{LSPS}}\left(i,j\right)/\left|I_{\text{LSPS}}\right|}{p_{i,j}\cdot g_{i,j}/\left|p_{\text{con}}\cdot g_{\text{max}}\right|},$$ (7)

where |⋅| denotes the sum over all matrix elements. *p*_con_ and *g*_max_ are then multiplied element-wise by $\sqrt{s^{I}}$, such that *p*_con_ ⋅ *g*_max_ agrees with *I*_LSPS_. The scaled values of all parameters are given in Table 3. The average connection strength (as defined by *p*_con_ ⋅ *g*_max_) between pyramidal cells and interneurons in the different stripes and layers is coded by the arrow width in Fig 2A.

**Simulation details.** All simulations were performed in customized C code written by the authors. Differential equations were numerically integrated using a 2^nd^-order Runge-Kutta method with a maximum time step of 0.05 ms, and all spikes, synaptic, and external events were exactly timed by adjusting the time steps accordingly. More specifically, whenever an incoming spike or a change in external currents occurs within the default time step, the time step is reduced accordingly and all equations are updated at the precise time of that event. Neurons were initialized with $V^{i}\left(0\right)=E_{L}^{i}$ and *w*^*i*^(0) = 0 for all *i*. MATLAB-based routines were used for parameter estimation and network analysis. All software is publicly available at https://www.zi-mannheim.de/index.php?id=626 and on the freely available repository ModelDB (http://senselab.med.yale.edu/ModelDB/).

### Estimation of background currents

The constant background currents *I* used in the simulations are assumed to replace missing synaptic input from the surrounding network not explicitly simulated. A network of sufficient size should be able to produce these amounts of current inherently (with physiologically realistic synaptic efficacies as used here) and thus self-sustain its *in vivo*-like activity. To test this idea, we computed the amount of current that is produced by a larger network *N* that is set up and connected exactly the same way as the actually simulated network *n* and compared it to the range of background currents that are required for *in vivo*-like activity. The currents *I*_*N*_ were modeled as the time-averaged synaptic currents that are elicited in a single neuron for each cell type (with the averaged cellular and synaptic parameters of all neurons of that type) in response to a bombardment of spikes drawn from a Poisson distribution that mimics a large number of input neurons, reflecting its input connectivity. For a Poisson input spike train with the same firing rate as in the original network, this yields the same synaptic current *I*_*n*_ as in the full simulation. Larger networks *N* are simulated by using a higher number of inputs, resulting in higher overall input spike rates. Because connection probabilities decay with distance between neurons [89], we independently tested two ways to increase the number of input neurons in the network: By increasing its spatial size *L* (measured in number of columns *N*_C_, each *L*_C_ = 300*μm* in diameter) or its within-column neuron density *D*. For cross-column input, we assumed a radial distribution of inputs [60, 89] and an exponential decay of connection probabilities with distance (*l*_*E*_ = 114*μm*, *l*_*I*_ = 95*μm*, see above). Only pyramidal cells in L2/3 as well as cross-column (IN-CC) and far-reaching (IN-F) interneurons project across columns. Specifically, the number of input neurons $N_{\text{syn}}^{i}$ projecting onto a neuron of cell type *i* is given by

$$\begin{array}{cc} & N_{\text{syn}}^{i}\left(D,L\right)=f_{C}^{i}\cdot N_{D}^{i}\left(0,L_{C}\right)+f_{N}^{i}\cdot N_{D}^{i}\left(L_{C},L\right)\\ \text{with}\; & N_{D}^{i}\left(L_{1},L_{2}\right)=2\pi D\cdot p_{\text{con}}^{i}\int_{L_{1}}^{L_{2}}x\cdot \exp(x/l_{i})dx. \end{array}$$ (8)

$p_{\text{con}}^{i}$ is the fraction of neurons that connects to cell type *i*. $N_{D}^{i}\left(L_{1},L_{2}\right)$ denotes the number of neurons within a hollow cylinder defined by the inner radius *L*_1_ and the outer radius *L*_2_ and the height of 1 mm [60] that are connected to a neuron of cell type *i* at the center of this cylinder, given a neuron density *D*. Thus, the two terms represent input from within the same column (up to *L*_C_) and from outside that column (up to the full radius *L*). The ratio of pyramidal cells and interneurons that project beyond a single column (Table 2) is reflected in different scaling factors for excitatory and inhibitory connectivity for input from within (*f*_C_) and from outside the same column (*f*_*N*_). The resulting background currents *I*_*N*_ are then computed independently for the four main cell types—pyramidal cells and interneurons in layer 2/3 and 5.

### Analysis of simulated and *in vivo* data

Two *in vivo* data sets were used for comparison with simulation results (kindly provided by Dr. Christopher Lapish, Indiana University Purdue University, Indianapolis and Dr. Thomas Hahn, Central Institute of Mental Health and BCCN Heidelberg-Mannheim). For spike trains and local field potentials, extracellular multiple single-unit recordings were obtained from the rat’s anterior cingulate cortex (ACC) while they were performing an eight-arm radial maze task [40]. Stationary periods (largely free from motor or sensory responses) were obtained from 381 units using a previously described stationarity-segmentation method [41]. For the voltage traces, we used patch-clamp recordings from anaesthetized rodents (see [109, 110] for details).

Spike trains, voltage traces and local field potentials from the network simulation and the *in vivo* data were analyzed the same way. For each model cell or recorded unit, mean and coefficient of variation (C_*V*_) of the interspike interval (ISI) distributions were computed. Autocorrelations of ISI series and the zero-lag cross-correlation CC(0) between ISIs from pairs of spike trains were computed as well according to the procedures described in [41] to correct for non-stationarities. All analyses were restricted to spike trains of at least 10 spikes to yield sensible estimates of single-cell statistics without cutting off too much of the low-rate tail from the distributions (see Results section for a more empirical justification).

Similarity among simulated and experimentally obtained distributions was assessed by two-sample Kolmogorov-Smirnov (KS) tests, where test statistic *D*_KS_ is bounded between zero (complete overlap) and one (maximally dissimilar distributions). Underlying distributions were inferred through kernel-density estimation [111] (implemented by the function “ksdensity” in MATLAB’s statistics toolbox). As KS test statistics may depend on sample size but different simulations vary in number of spikes, we limited the number of data points to a sufficiently low, common value (30), repeated KS tests with 100 random drawings, and report averages across obtained p values and KS statistics. The overall similarity of a simulation data set with the experimental spike data is quantified by conducting the test for the mean ISI, the C_*V*_ and the CC(0) distributions, and reporting the minimal p or the maximal *D*_KS_ value of those three (i.e., the value associated with the largest difference between the compared distributions).

Finally, we visualize the statistical overlap of distributions by plotting shaded areas representing the SEM around the mean at each value of the experimental distributions, which are computed from the 100 bootstrap samples as indicated above.
